# The long-term intercorrelation between post-burn pain, anxiety, and depression: a post hoc analysis of the “RE-ENERGIZE” double-blind, randomized, multicenter placebo-controlled trial

**DOI:** 10.1186/s13054-024-04873-8

**Published:** 2024-03-22

**Authors:** Adriana C. Panayi, Daren K. Heyland, Christian Stoppe, Marc G. Jeschke, Oliver Didzun, Dany Matar, Christian Tapking, Alen Palackic, Björn Bliesener, Leila Harhaus, Samuel Knoedler, Valentin Haug, Amir K. Bigdeli, Ulrich Kneser, Dennis P. Orgill, Gabriel Hundeshagen

**Affiliations:** 1 Department of Hand-, Plastic and Reconstructive Surgery, Burn Center, BG Trauma Center Ludwigshafen, University of Heidelberg, Ludwig-Guttmann-Straße 13, 67071 Ludwigshafen/Rhine, Germany; 2 Clinical Evaluation Research Unit, Department of Critical Care Medicine, Queen’s University, Kingston, ON Canada; 3 Department of Anaesthesiology, Intensive Care, Emergency and Pain Medicine, University Hospital, Würzburg, Würzburg, Germany; 4 Department of Cardiac Anesthesiology and Intensive Care Medicine, Charité Berlin, Berlin, Germany; 5 Hamilton Health Sciences Research, Department of Surgery, McMaster University, Hamilton, ON Canada; 6 Division of Plastic Surgery, Department of Surgery, Brigham and Women’S Hospital, Harvard Medical School, Boston, MA USA; 7 The Johns Hopkins University School of Medicine, Baltimore, MD USA

**Keywords:** Burn injury, Quality of life, Pain, Anxiety, Depression

## Abstract

**Background:**

Despite the growing prevalence of burn survivors, a gap persists in our understanding of the correlation between acute burn trauma and the long-term impact on psychosocial health. This study set out to investigate the prevalence of long-term pain and symptoms of anxiety and depression in survivors of extensive burns, comparing this to the general population, and identify injury and demographic-related factors predisposing individuals to psychosocial compromise.

**Methods:**

RE-ENERGIZE was an international, double-blinded, randomized-controlled trial that enrolled 1200 patients with partial- or full-thickness burns that required surgical treatment. For the post hoc analysis, we excluded participants who did not complete the Short Form Health Survey (SF-36) questionnaire. Normative data were taken from the 2021 National Health Interview Survey dataset. Propensity score matching was performed using the nearest-neighbor 1-to-1 method, and the two cohorts were compared in terms of chronic pain, and symptoms of anxiety and depression. A multivariable analysis was performed on the burns cohort to identify factors predicting post-discharge pain and symptoms of anxiety and depression.

**Results:**

A total of 600 burn patients and 26,666 general population adults were included in this study. Following propensity score matching, both groups comprised 478 participants each, who were predominately male, white, overweight and between 20 and 60 years old. Compared to the general population, burn patients were significantly more likely to report the presence of moderate and a lot of pain (*p* = 0.002). Symptoms of anxiety were significantly higher in the burn population in two of four levels (most of the time; some of the time; *p* < 0.0001 for both). Responders in the burn population were significantly less likely to report the absence of depressive symptoms (*p* < 0.0001). Burn patients were also significantly more likely to report that their mental health affects their social life. TBSA, history of depression, and female sex were identified as independently associated factors for pain, anxiety, and depressive symptoms. The presence of chronic pain and anxiety symptoms independently predicted for symptoms of depression.

**Conclusions:**

Analyzing the largest multicenter cohort of patients with extensive burns, we find that burn injury is associated with chronic pain, and symptoms of anxiety and depression. In addition, TBSA-burned and history of depression directly correlate with the prevalence of chronic pain, and symptoms of anxiety and depression. Finally, pain, and symptoms of anxiety and depression are interrelated and may have interactive effects on the process of recovery following burn injury. Burn patients would, therefore, benefit from a multidisciplinary team approach with early mobilization of pain and mental health experts, in order to promptly prevent the development of psychosocial challenges and their consequences.

**Supplementary Information:**

The online version contains supplementary material available at 10.1186/s13054-024-04873-8.

## Introduction

In his seminal 2005 perspective, "Burn treatment's evolution in the twentieth century," John F. Burke illustrated the commendable strides made in reducing burn-related mortality, prompting a paradigmatic shift toward prioritizing long-term outcomes and elevating the quality of life of survivors [1]. Yet, nearly two decades have passed and to this day a critical gap persists in our understanding of the correlation between burn trauma and its consequential impact on both physical and psychosocial functions long-term.

Severe burn injuries are among the most complex forms of trauma and are characterized by their acute, devastating, topical and systemic nature [2]. The treatment of severe burns is at the intersection of acute surgery, to excise and resurface damaged tissue [3], and specialized intensive care, that aims to manage acute shock, systemic hyperinflammation, catabolic hypermetabolism, severe immunosuppression and complications like infection and sepsis [4, 5], which are main drivers of morbidity and mortality. Despite these challenges, there has been a continuous and steady improvement in survival rates of severe and even catastrophic burn injuries in recent decades [6], which is attributed to advances in specialized burn care, research and understanding of acute pathophysiology, as well as intensive care in general. A never-before-seen cohort of survivors forces research to refocus away from incremental reduction of acute mortality and on to long-term outcomes and quality of life [7].

Therefore, Burke and others argue that with increasing survival rates among burn patients, addressing the psychological needs of survivors becomes paramount for achieving a quality of life close to pre-burn levels and successful reintegration into society. The effect of burn injuries patients’ mental well-being is somewhat known but poorly understood. Several studies revealed a significantly increased prevalence of depression, anxiety, post-traumatic stress disorder (PTSD), concerns about body disfigurement, social isolation, and financial burdens after burn injury [8–17]. Such challenges persist, with a recent meta-analysis finding that survivors of severe burn injury fail to reach normal levels of key quality of life indicators even four years after the injury [18].

Various factors exacerbate post-burn psychological symptoms, including the depth and percentage of total body surface area (TBSA) burned, pain intensity and the invasive nature of surgical and intensive care interventions [9–11]. Biological mechanisms have also been implicated including associations with the level of cortisol and alpha-amylase [19]. The persistent pain endured by burn survivors—which can range from pruritus to joint pain to chronic, neuropathic pain—extends beyond its hindrance to the healing process; it permeates their lives, diminishing the overall quality of life. Depressive symptoms within the first year post-injury vary widely, from 4% at discharge to 10–23% one year after injury [14, 15, 17] which are well above the general population rate at for both men (3–5%) and women (8–10%) [20]. One study extending beyond the first year after burn injury indicates stable depression levels over a two-year period, but heightened pain and compromised peer relationships and physical functioning [21].

The current evidence, however, stems from small monocentric studies, often without a normative comparison group. Such small-scale, single center studies suffer from limited generalizability, reproducibility, and validity of clinical translation. Debate, therefore, still exists on the different factors that can predispose to pain, depression and anxiety post-discharge, as well as on the time-course and potential prevalence of these psychosocial challenges.

The primary objective of this study was to utilize one of the largest multicenter cohort of patients with extensive burns to determine the prevalence of pain, and symptoms of anxiety and depression and compare this to a non-burned general representative population. The hypothesis is that burn survivors are more likely to experience chronic pain, and symptoms of depression and anxiety. Additionally, the study aims to identify demographic and burn-related factors predisposing individuals to chronic pain, and symptoms of anxiety and depression, thereby informing clinical practices, research initiatives, and policymaking efforts.

## Methods

### Data source

Patients were identified from the previously published Randomized Trial of Enteral Glutamine to Minimize the Effects of Burn Injury (RE-ENERGIZE) trial. Briefly, the RE-ENERGIZE study was an international, double-blinded, randomized-controlled trial evaluating the use of supplemental enteral glutamine on time-to-discharge alive in patients with severe burn injuries.

Severe burn injury was defined as partial- or full-thickness burns necessitating surgical treatment, while eligible total body surface area burned (TBSA) varied: > 20% in patients 18 to 39 years of age, > 15% in the presence of concomitant inhalation injury; > 15% in patients 40 to 59 years of age; and > 10% in patients 60 years of age or older. Besides the administration of either 0.5 g (per kg BW) enteral glutamine or placebo per day, all other clinical decisions were left to the discretion of the clinical team.

Data collected included patient demographics and characteristics (age; sex; ethnicity; body mass index/BMI; comorbidities; alcohol and smoking status), injury characteristics (TBSA; cause of burn: scald, fire, chemical, radiation, other; TBSA burned; inhalation injury; APACHE-II score), in-hospital care (duration of mechanical ventilation) and outcomes (ICU length of stay in days; hospital length of stay/LOHS in days; discharge destination). Ethnicity was based on self-dentification from previously defined categories (White/Caucasian, Black/African American, Hispanic, Asian/Pacific Islander, and Native). Data on the participating burn centers were also collected (geographic region and characteristics). Among the comorbidities collected on admission to hospital were psychological conditions registered as the presence of anxiety/panic disorders and the presence of depression.

Post-discharge the patients were contacted to complete the 36-Item Short Form Health Survey (SF-36) questionnaire. The date of completion of each questionnaire was also collected allowing for calculation of the time from admission to long-term follow-up.

### Normative data source

The data for the general population were taken from the publicly available 2021 National Health Interview Survey (NHIS) dataset. The NHIS is administered by the National Center for Health Statistics (NCHS) annually since 1957 through telephone or face-to-face (household) interviews to collect cross-sectional data to assess the health status of the US population. The content and structure of the dataset was redesigned in 2019 and differs from its previous questionnaire design (1997–2018). A multistage probability study design is used resulting in data that are nationally representative of households and non-institutionalized civilian US populations. Data collection is continuous from January to December each year. The NHIS includes residents of households and non-institutional group quarters such as homeless shelters, rooming houses, and group homes. Black, Hispanic, and Asian populations are oversampled [22]. Subjects hospitalized overnight over the past year (i.e., subjects who responded yes to the question: During the past 12 months, have you been hospitalized overnight?) were excluded from this analysis, therefore, indirectly excluding any patients who might have been hospitalized for burn injury, as well as other acute or chronic ailments from the normative data sample.

### Outcomes

The primary outcomes were chronic pain, and symptoms of anxiety and depression, from hereon referred to as pain, anxiety, and depression, as recorded based on questionnaires (SF-36 or variations thereof). It should be clarified that the SF-36 is a general questionnaire that provides insight into symptoms of depression and anxiety. Although the terms anxiety and depression are used throughout the manuscript, the respondents where not officially diagnosed with anxiety and depression based on the Diagnostic and Statistical Manual of Mental Disorders. The responses for the SF-36 and the NHIS questions pertaining to pain, anxiety and depression were compared, adjusting the format accordingly. The questions can be found in Table 1.

**Table 1:** Format of questions as presented to the burn population (SF-36 questionnaire) and the general population (NHIS questionnaire)

Outcome	SF-36 questionnaire	NHIS questionnaire
Pain	How much bodily pain have you had during the past 4 weeks?	Thinking about the last time you had pain, how much pain did you have?
Pain impact on work	During the past 4 weeks, how much did pain interfere with your normal work (including both work outside the home and housework)?	Over the past three months, how often did your pain limit your life or work activities? Would you say never, some days, most days, or every day?
Anxiety	How much of the time during the past 4 weeks have you been very nervous?	During the past 30 days, how often did you feel nervous?
Depression (1)	How much of the time during the past 4 weeks have you felt so down in the dumps that nothing could cheer you up?	During the past 30 days, how often did you feel so sad that nothing could cheer you up?
Depression (2)	How much of the time during the past 4 weeks have you felt downhearted and depressed?	During the past 30 days, how often do you feel depressed?
Anxiety and depression impact on social life	During the past 4 weeks, how much of the time has your physical health or emotional problems interfered with your social activities (like visiting with friends, relatives, etc.)?	Because of a physical, mental, or emotional condition, do you have difficulty participating in social activities such as visiting friends, attending clubs and meetings, or going to parties?

*SF-36* 36-Item Short Form Health Survey; *NHIS* National Health Interview Survey

### Statistical analysis

The data from the NHIS and RE-ENERGIZE databases were collected and equalized in Microsoft Excel® 2020 (Microsoft, Redmond, WA, USA). Categorical data are presented as absolute numbers (*n*) and percentages (%) and continuous data as averages and standard deviations. Categorical variables are compared using a Chi-square or Fishers exact test, as appropriate. Continuous variables are compared using a Student’s T-test. R statistical software (version 4.1.2) was used to perform propensity score matching (Matchlt package) with nearest-neighbor 1-to-1 matching, whereby the treated unit, “burn patient,” is matched to a control, “general population,” in terms of a distance measure such as a logit (method = “nearest”). The quality of the matching can be seen in the histograms and jitter plots in Additional file 1: Figs. S1 and S2. Patients were matched for any variable found to be significant during descriptive comparison of the demographics and characteristic of the two cohorts. The variables that were matched were sex, age, ethnicity, BMI, the presence of hypertension, asthma, renal disease, cancer, arthritis, history of anxiety or depression, and current smoker. A *p*-value < 0.05 was considered statistically significant. The matched cohort was then used to compare the primary outcomes. As the normative comparison group is limited to the USA, a further propensity score matching analysis was performed, as above, but including only burn patients treated in US institutions. A multivariable analysis was performed on the burns cohort to identify independently associated factors for post-discharge pain, depression, and anxiety. Variables included were alcohol abuse, smoking, type of burn, BMI, TBSA, LOHS, history of anxiety or panic disorder, history of depression, sex, age, and ethnicity. A further multivariable analysis was performed on the burns cohort including post-discharge pain and anxiety with the aforementioned variables to establish their independent relationship with depression. The demographics and characteristics of the SF-36 responders and non-responders were compared using a Fishers exact test or Student’s T-test, as appropriate, to establish whether the results were representative of the entire burn cohort. Finally, the responders were stratified according to whether they received glutamine or placebo and the outcomes were compared using a Fishers exact test to establish the effect of glutamine. All analyses were conducted in GraphPad Prism (version 9). Data visualization was performed in GraphPad Prism and Illustrator.

## Results

### Cohort demographics and characteristics

**Fig. 1:**
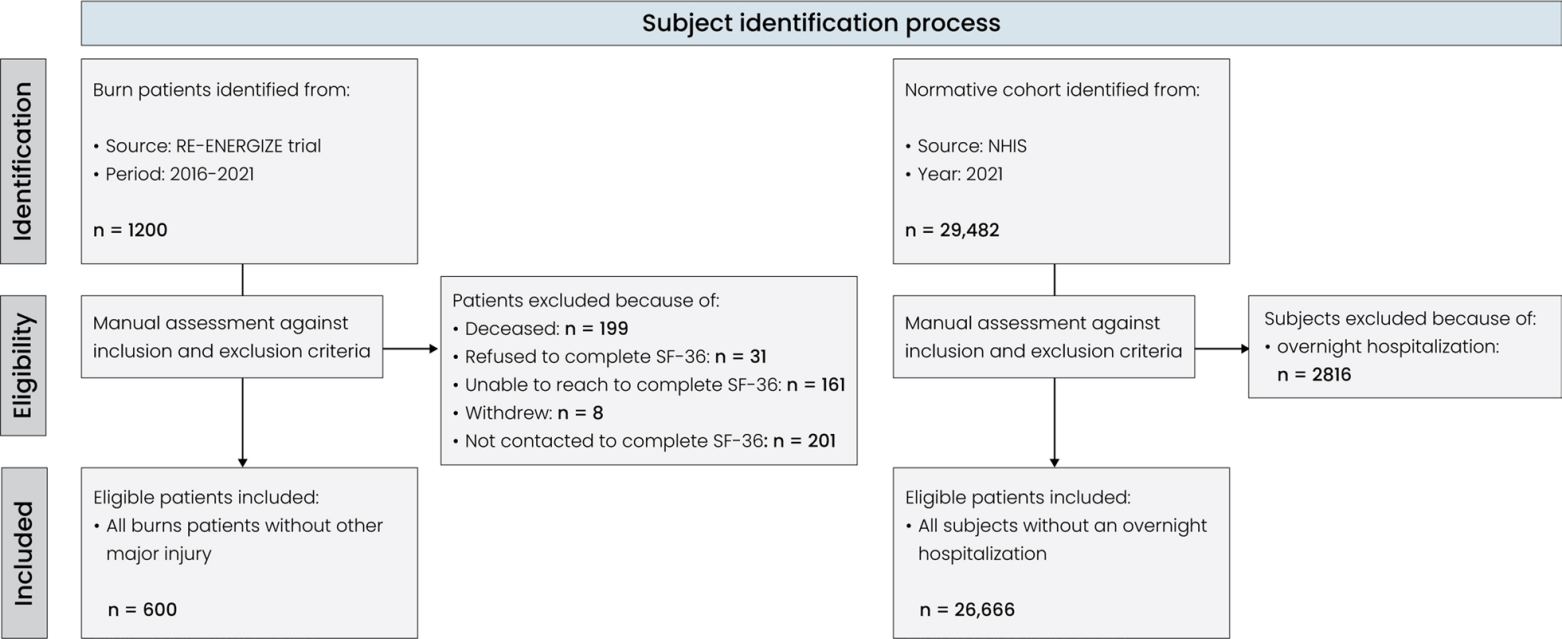
Subject selection process

Of a total of 1200 burn patients, 600 completed the necessary questionnaires and were therefore included in this study. 199 participants were reported as deceased, while 401 refused to complete the questionnaire or withdrew from the trial. A total of 26,666 general population adults were identified. The subject selection process is visualized in Fig. 1. The majority of the burn population was male (73.5%), as opposed to an equal distribution in the normative group (male = 45.9%; Table 2). The burn cohort was significantly younger than the normative population (48.7 ± 17.1 vs. 52.0 ± 18.3; *p* < 0.0001) and had a significantly lower BMI (28.3 ± 6.0 vs. 33.7 ± 23.1; *p* < 0.0001). White and Caucasian was the predominant racial group in both cohorts (B: 76.0% vs. N: 74.4%), although the burn cohort had a higher percentage of native (3.0% vs. 0.8%) and Hispanic (8.3% vs. 0.2%) subjects, while the normative cohort had a higher percentage of Black or African American subjects (10.8% vs. 6.3%). Comparison of the 600 respondents to the 401 non-responders established that non-responders were on average younger (*p* = 0.001), less likely to be Hispanic (*p* = 0.04) and more likely to be African American (*p* = 0.02), more likely to be smokers (*p* = 0.01), more likely to have had inhalation injury (*p* < 0.0001), more likely to have been transferred to a ward in another hospital (*p* = 0.03), and had on average shorter hospital (*p* = 0.01) and ICU stays (*p* = 0.03; Additional file 1: Table S1).

**Table 2:** Subject demographics and characteristics

	Burn (*n* = 600)	Normative (*n* = 26,666)	*p* value
Sex			< 0.0001
Male	441 (73.5)	12,244 (45.9)	
Female	159 (26.5)	14,420 (54.1)	
Age in years, M (SD)	48.7 (17.1)	52.0 (18.3)	< 0.0001
Age group (years)			0.0017
Adolescent (18–19)	11 (1.8)	448 (1.7)	
Adults (20–60)	413 (68.8)	16,508 (61.9)	
Older adults (> 60)	176 (29.3)	9710 (36.4)	
BMI in kg/m^2^, M (SD)	28.3 (6.0)	33.7 (23.1)	< 0.0001
Country US	311 (51.8)	26,666 (100.0)	< 0.0001
Ethnicity			< 0.0001
White or Caucasian	456 (76.0)	19,849 (74.4)	
Native	18 (3.0)	200 (0.8)	
Hispanic	50 (8.3)	64 (0.2)	
Black or African American	38 (6.3)	2879 (10.8)	
Asian or Pacific Islander	31 (5.2)	1733 (6.5)	
Comorbidities			
Angina	4 (0.7)	392 (1.5)	0.10
Myocardial infarction	14 (2.3)	799 (3.0)	0.35
Hypertension	150 (25.0)	9109 (34.2)	< 0.0001
Stroke	13 (2.2)	732 (2.7)	0.39
COPD	20 (3.3)	1316 (4.9)	0.07
Asthma	26 (4.3)	3575 (13.4)	< 0.0001
Dementia	4 (0.7)	276 (1.0)	0.38
Diabetes	72 (12.0)	2565 (9.6)	0.05
Obesity	66 (11.0)	9539 (35.8)	< 0.0001
Moderate/severe renal disease	4 (0.7)	737 (2.8)	0.002
Cancer	27 (4.5)	3033 (11.4)	< 0.0001
Lymphoma	0 (0.0)	98 (0.4)	0.14
Leukemia	1 (0.2)	39 (0.1)	0.90
Arthritis	19 (3.2)	6442 (24.2)	< 0.0001
Smoking (current)	170 (28.3)	3064 (11.5)	< 0.0001
Mental health			
Anxiety	46 (7.7)	4234 (15.9)	< 0.0001
Depression	58 (9.7)	4627 (17.4)	< 0.0001

Reported as *n* (%), unless otherwise stated

*BMI* body mass index; *M* mean; *SD* standard deviation; *n* number

In terms of comorbidities, the burn cohort was significantly less likely to have hypertension (25.0% vs. 34.2%; *p* < 0.0001), asthma (4.3% vs. 13.4%; *p* < 0.0001), obesity (11% vs. 35.8%; *p* < 0.0001), renal disease (0.7% vs. 2.8%; *p* = 0.002), cancer (4.5% vs. 11.4%; *p* < 0.0001) and arthritis (3.2% vs. 24.2%; *p* < 0.0001). Burn patients were significantly more likely to be smokers (28.3% vs. 11.5%; *p* < 0.0001). A history of both anxiety and depression was significantly more likely in the normative population (Anxiety: 7.7% vs. 15.9%; Depression: 9.7% vs. 17.4%; *p* < 0.0001). The majority of the burn cohort data was from North America (US: 51.8% and Canada: 19.5%). All data for the normative cohort were US data.

### Burn-specific characteristics and outcomes

The mean TBSA was 30.6% (± 15.1%; Table 3). The main cause of burn was fire (89.3%) followed by scalding (7.5%). Approximately half of the patients received glutamine (49%) and ventilation (53.5%) during their hospital stay. None of the patients suffered from inhalation injury. The mean APACHE-II score was 13.5 (± 7.9) and the mean SOFA score 2.9 (± 2.8). Mean questionnaire follow-up was approximately five months after discharge (164 ± 68 days). The mean length of ICU stay was 35.0 days (± 21.8), and the mean length of hospital stay was 37.4 days (± 21.8). Half of the patients were discharged home (50.0%) and a third to a rehabilitation unit (31.8%).

**Table 3:** Burn cohort characteristics and outcomes

	Burn (*n* = 600)
TBSA%, M (SD)	30.6 (15.1)
Questionnaire follow-up in days, M (SD)	164.0 (68.0)
Country	
US	311 (51.8)
Austria	2 (0.3)
Belgium	21 (3.5)
Brazil	2 (0.3)
Canada	117 (19.5)
Germany	21 (3.5)
Italy	17 (2.8)
Paraguay	21 (3.5)
Singapore	2 (2.8)
Spain	8 (1.3)
Sweden	8 (1.3)
Thailand	14 (2.3)
UK	56 (9.3)
SOFA score	2.9 (2.8)
APACHE-II score	13.5 (7.9)
Received ventilation	321 (53.5)
Received glutamine	294 (49.0)
Cause of burn	
Chemical	15 (2.5)
Fire	536 (89.3)
Scald	45 (7.5)
Other	4 (0.7)
Inhalation injury	0 (0.0)
Discharge destination	
Home	300 (50.0)
Rehabilitation unit	191 (31.8)
Long-term care facility	26 (4.3)
Ward in another hospital	16 (2.7)
ACU in another hospital	9 (1.5)
Length of hospital stay in days, M (SD)	42.2 (24.9)
Length ICU stay in days, M (SD)	38.7 (24.3)

Reported as *n* (%), unless otherwise stated

*M* mean; *SD* standard deviation; *n* number; *TBSA* total body surface area; *ICU* intensive care unit; *ACU* acute care unit

### Group composition after propensity score matching

Following propensity score matching, both groups comprised mainly male (∼70%), white subjects (∼70%), between 20 and 60 years of age (∼66%), with a mean BMI within the overweight range (∼28 kg/m^2^). The normative population had significantly more black or African American subjects (1.21% vs. 5.9%; Table 3). The two groups were matched in terms of all comorbidities that significantly differed in Table 2.

### Pain, anxiety, and depression in the propensity score-matched cohorts

**Fig. 2:**
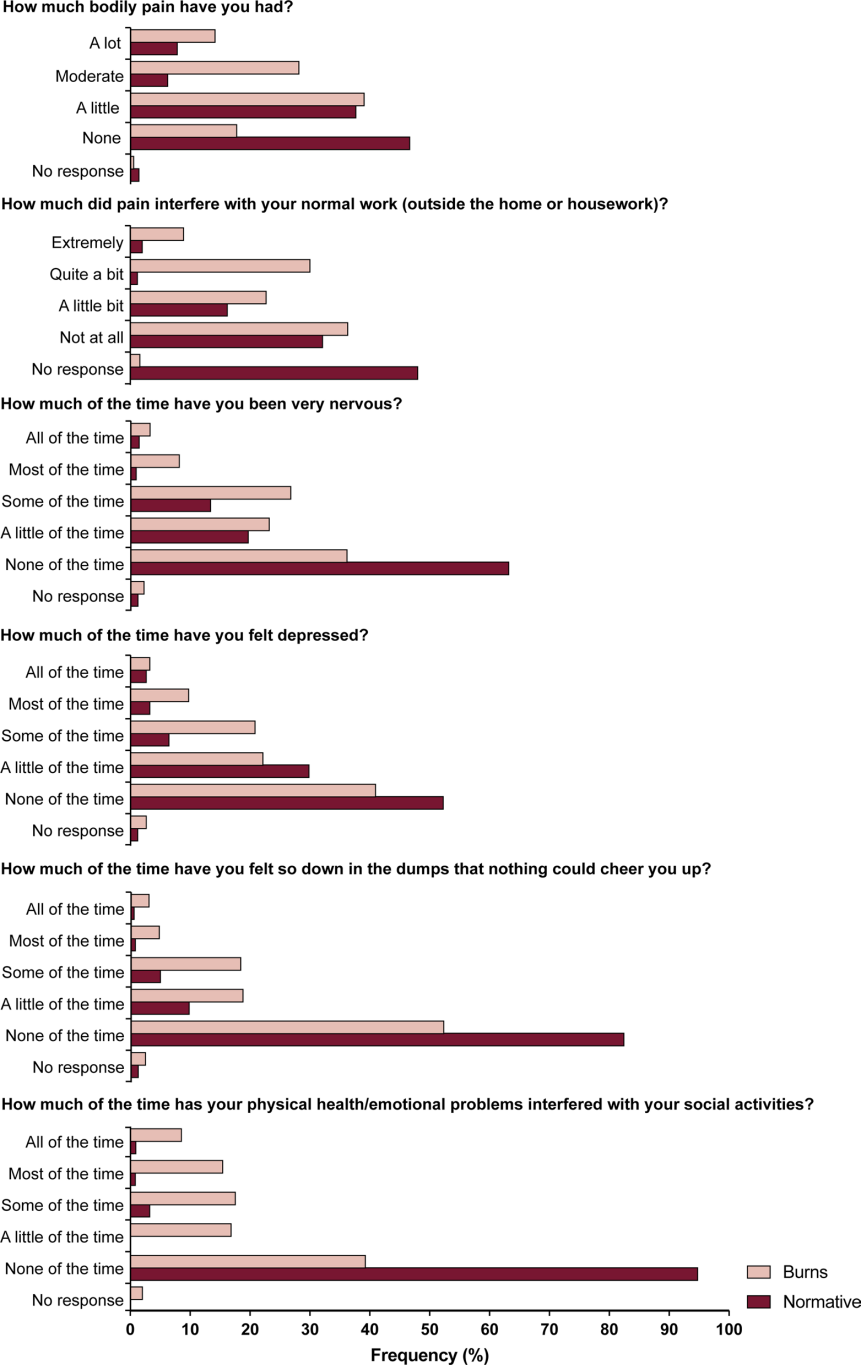
Response rates in the propensity score-matched groups. Exact numbers and significance rates can be seen in Table 4

Compared to the general population, patients with a burn injury were significantly less likely to report total absence of pain (17.8% vs. 46.7%; *p* < 0.0001) and significantly more likely to report the presence of moderate (28.2% vs. 6.3%; *p* < 0.0001) or a lot of pain (14.2% vs. 7.9%; *p* = 0.002; Table 4; Fig. 2).

**Table 4:** Subject demographics, characteristics and outcomes following propensity score matching

	Burn (*n* = 478)	Normative (*n* = 478)	*p* value
Sex (male)	347 (72.6)	345 (72.2)	0.89
Age in years, M (SD)	49.5 (17.4)	49.1 (17.5)	0.73
Age group (years)			
Adolescent (18–19)	9 (1.9)	9 (1.9)	> 0.99
Adults (20–60)	317 (66.3)	331 (69.2)	0.37
Older adults (> 60)	157 (32.8)	138 (28.9)	0.21
BMI in kg/m^2^, M (SD)	28.1 (6.0)	28.3 (9.1)	0.72
Ethnicity			
White or Caucasian	363 (75.9)	330 (69.0)	0.02
Native	13 (2.7)	5 (1.0)	0.09
Hispanic	39 (8.2)	32 (6.7)	0.46
Black or African American	28 (5.9)	58 (12.1)	0.001
Asian or Pacific Islander	30 (6.3)	45 (9.4)	0.09
Other	5 (1.0)	8 (1.7)	0.58
How much bodily pain have you had?			
None	85 (17.8)	223 (46.7)	< 0.0001
A little	187 (39.1)	180 (37.7)	0.64
Moderate	135 (28.2)	30 (6.3)	< 0.0001
A lot	68 (14.2)	38 (7.9)	0.002
No response	3 (0.6)	7 (1.5)	0.34
How much did pain interfere with your normal work (outside the home or housework)?			
Not at all	174 (36.4)	154 (32.2)	0.17
A little bit	109 (22.8)	78 (16.3)	0.01
Quite a bit	144 (30.1)	6 (1.3)	< 0.0001
Extremely	43 (9.0)	10 (2.1)	< 0.0001
No response	8 (1.7)	230 (48.1)	< 0.0001
How much of the time have you been very nervous?			
All of the time	16 (3.3)	7 (1.5)	0.09
Most of the time	39 (8.2)	5 (1.0)	< 0.0001
Some of the time	128 (26.8)	64 (13.4)	< 0.0001
A little of the time	111 (23.2)	94 (19.7)	0.18
None of the time	173 (36.2)	302 (63.2)	< 0.0001
No response	11 (2.3)	6 (1.3)	0.33
How much of the time have you felt depressed?			
All of the time	16 (3.3)	13 (2.7)	0.57
Most of the time	47 (9.8)	16 (3.3)	< 0.0001
Some of the time	100 (20.9)	31 (6.5)	< 0.0001
A little of the time	106 (22.2)	143 (29.9)	0.006
None of the time	196 (41.0)	269 (52.3)	< 0.0001
No response	13 (2.7)	6 (1.3)	0.16
How much of the time have you felt so down in the dumps that nothing could cheer you up?			
All of the time	15 (3.1)	3 (0.6)	0.007
Most of the time	23 (4.8)	4 (0.8)	0.0003
Some of the time	88 (18.4)	24 (5.0)	< 0.0001
A little of the time	90 (18.8)	47 (9.8)	< 0.0001
None of the time	250 (52.3)	394 (82.4)	< 0.0001
No response	12 (2.5)	6 (1.3)	0.23
How much of the time has your physical health/emotional problems interfered with your social activities?			
All of the time	41 (8.6)	5 (1.0)	< 0.0001
Most of the time	74 (15.5)	4 (0.9)	< 0.0001
Some of the time	84 (17.6)	16 (3.3)	< 0.0001
A little of the time	81 (16.9)	0 (0.0)	< 0.0001
None of the time	188 (39.3)	453 (94.8)	< 0.0001
No response	10 (2.1)	0 (0.0)	0.002

Reported as *n* (%), unless otherwise stated

*BMI* body mass index; *M* mean; *SD* standard deviation; *n* number

Subjects in the burn population were significantly more likely to report that pain has some interference on their work, with 22.8% reporting a little bit (vs. N: 16.3%; *p* = 0.01), 30.1% quite a bit (vs. N: 1.3%; *p* < 0.0001), and 9% extremely (vs. N: 2.1%; *p* < 0.0001). Subjects in the normative population who had reported the absence of pain gave no response to the question about the impact of pain on their normal work, hence the high prevalence of no response (48.1%).

Anxiety, indirectly measured by the question “How much of the time have you been nervous?” was significantly higher in the burn population in two of four levels (most of the time and some of the time; *p* < 0.0001 for both). The absence of nervousness was significantly lower in the burn population (36.2% vs. 63.2%; *p* < 0.0001).

When asked how much of the time they felt depressed, responders in the burn population were significantly more likely to report most of the time, some of the time and a little of the time, while significantly less likely to report the absence of depression (41.0% vs. 52.3%; *p* < 0.0001). The level of depression was assessed with the second question “How much of the time have you felt so down in the dumps that nothing could cheer you up?”, for which burn patients were significantly more likely to answer all, most, some and a little of the time, while significantly less likely to answer none of the time. Burn patients were also significantly more likely to report that their physical and emotional health affects their social life in all four response levels (all, most, some and a little of the time), and less likely to answer none of the time.

A second propensity score matching analysis was performed including only data of burn patients treated in the USA and verified the results of the aforementioned propensity score matching (Additional file 1: Table S2). The response rates to the questions from the entire burn and general population cohorts are shown in Additional file 1: Tables S3 and S4.

### Factors predisposing to pain, anxiety, and depression in the Burn cohort

**Fig. 3:**
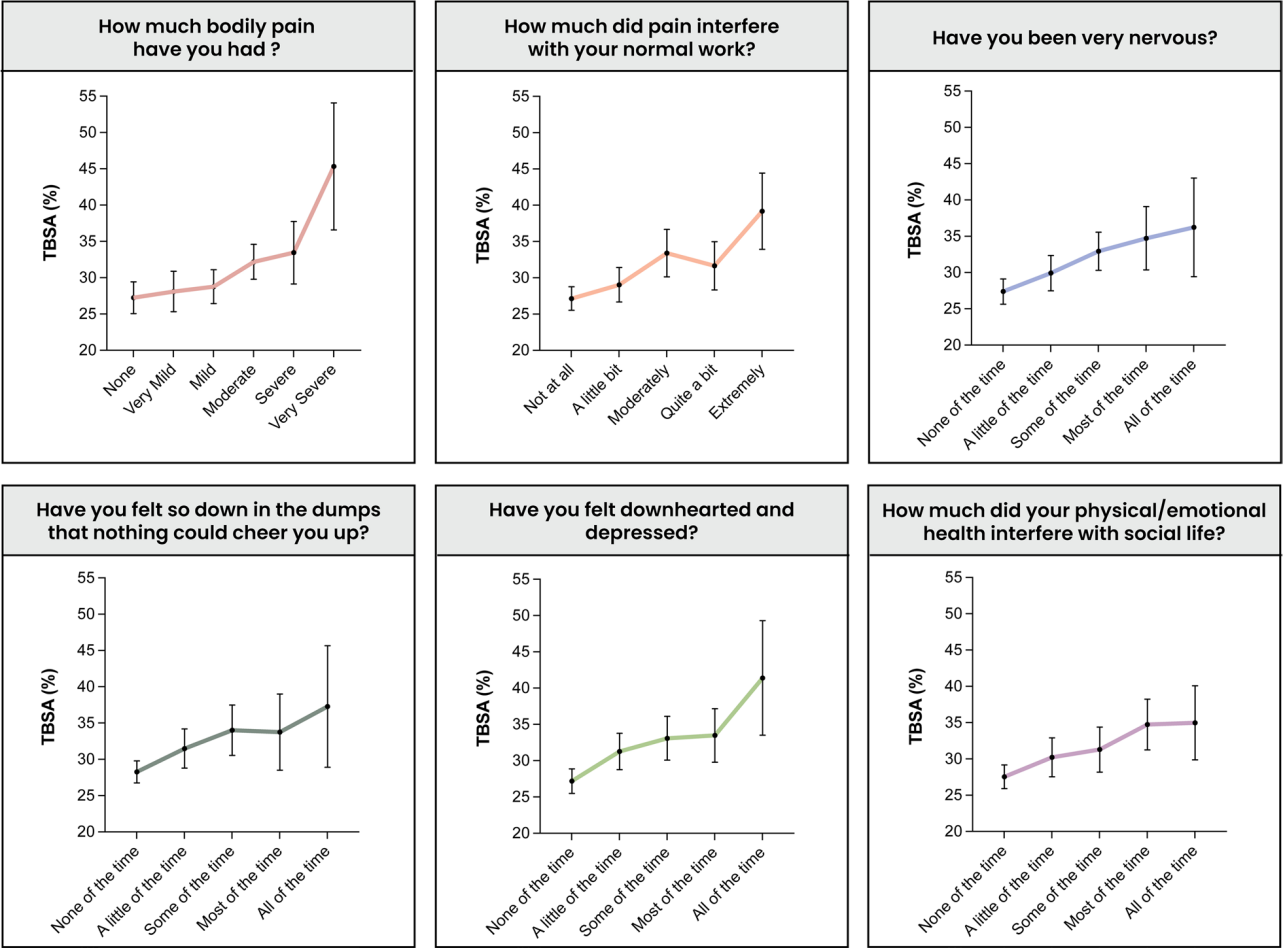
Response rates against TBSA

A multivariable linear regression was performed using the entire burn cohort to identify associated factors for pain, anxiety, and depression (Table 5). Smoking, BMI, TBSA, LOHS, history of depression, female sex, age and black or African American ethnicity were identified as independent associated factors for pain. Smoking, TBSA, history of depression, and female sex were identified as independent associated factors for depression. TBSA, history of depression, and female sex were identified as independent associated factors for anxiety. A correlation between TBSA and severity of pain, anxiety and depression was noted across all assessments (Fig. 3). The effect of glutamine on the primary outcomes was also investigated (Additional file 1: Table S5) identifying two significant differences: glutamine recipients were less likely to respond they felt depressed “a little of the time,” while more likely to respond they felt nervous “some of the time.”

**Table 5:** Multivariate assessment of pain, anxiety and depression

Primary outcomes	Estimate	95% CI	*p*-value
Pain			
Alcohol abuse	− 0.01	− 0.38–0.37	0.98
Smoking	0.37	0.10–0.64	**0.01**
Burn (Scald)	− 0.01	− 0.44–0.41	0.95
Burn (Chemical)	− 0.62	− 1.32–0.09	0.09
Burn (Other)	1.24	− 0.06–2.55	0.06
BMI	0.02	0.00–0.04	**0.03**
TBSA (%)	0.02	0.01–0.03	**0.003**
LOHS	0.01	0.00–0.02	**0.01**
History of anxiety or panic disorder	− 0.33	− 0.81–0.16	0.18
History of depression	0.43	0.01–0.85	**0.04**
Sex (female)	0.51	0.24–0.77	**0.0002**
Age	0.01	0.00–0.02	**0.02**
Ethnicity (Native)	− 0.07	− 0.76–0.62	0.85
Ethnicity (Black or African American)	0.77	0.31–1.229	**0.001**
Ethnicity (Hispanic)	− 0.34	− 0.73–0.06	0.09
Ethnicity (Asian or Pacific Islander)	0.50	− 0.02–1.01	0.06
Ethnicity (Other)	0.87	− 0.19–1.94	0.11
Depression			
Alcohol abuse	− 0.05	− 0.38–0.27	0.75
Smoking	0.29	0.06–0.53	**0.02**
Burn (Scald)	− 0.12	− 0.49–0.25	0.52
Burn (Chemical)	0.10	− 0.55–0.75	0.76
Burn (Other)	0.09	− 1.03–1.22	0.87
BMI	0.00	− 0.01–0.02	0.72
TBSA (%)	0.01	0.01–0.02	**0.002**
LOHS	0.00	0.00–0.01	0.47
History of anxiety or panic disorder	0.04	− 0.38–0.46	0.86
History of depression	0.67	0.31–1.04	**0.0004**
Sex (female)	0.31	0.08–0.54	**0.01**
Age	0.00	− 0.01–0.00	0.27
Ethnicity (Native)	0.11	− 0.48–0.70	0.72
Ethnicity (Black or African American)	0.33	− 0.07–0.73	0.11
Ethnicity (Hispanic)	0.00	− 0.34–0.35	0.98
Ethnicity (Asian or Pacific Islander)	0.03	− 0.42–0.48	0.90
Ethnicity (Other)	0.44	− 0.48–1.36	0.34
Anxiety			
Alcohol abuse	0.29	− 0.04–0.62	0.08
Smoking	0.20	− 0.04–0.44	0.10
Burn (Scald)	− 0.09	− 0.45–0.28	0.64
Burn (Chemical)	− 0.06	− 0.71–0.59	0.86
Burn (Other)	0.16	− 0.97–1.28	0.79
BMI	0.00	− 0.01–0.02	0.65
TBSA (%)	0.01	0.00–0.02	**0.004**
LOHS	0.00	0.00–0.01	0.67
History of anxiety or panic disorder	0.18	− 0.24–0.61	0.40
History of depression	0.39	0.02–0.76	**0.04**
Sex (female)	0.37	0.14–0.60	**0.001**
Age	0.00	− 0.01–0.00	0.31
Ethnicity (Native)	0.38	− 0.21–0.98	0.20
Ethnicity (Black or African American)	0.22	− 0.18–0.63	0.28
Ethnicity (Hispanic)	− 0.13	− 0.47–0.22	0.47
Ethnicity (Asian or Pacific Islander)	0.18	− 0.26–0.62	0.43
Ethnicity (Other)	0.18	− 0.74–1.10	0.70

All variables included in the analysis are shown

*TBSA* total body surface area; *BMI* body mass index; *LOHS* length of hospital stay; *M* mean; *SD* standard deviation; *n* number

Significant p values highlighted in bold font

### The interrelationship between pain, anxiety, and depression in the Burn cohort

A multivariable linear regression was also performed using the entire burn cohort to identify the interrelationship between depression, pain and anxiety (Table 6). The presence of pain and anxiety and a history of depression were identified as independent associated factors for depression.

**Table 6:** Multivariate assessment of depression, when pain and anxiety are included as variables. All variables included in the analysis are shown

Depression	Estimate	95% CI	*p*-value
Pain	0.57	0.50–0.63	** < 0.0001**
Anxiety	0.21	0.15–0.27	** < 0.0001**
Alcohol abuse	− 0.18	− 0.42–0.06	0.14
Smoking	0.09	− 0.09–0.26	0.33
Burn (Scald)	− 0.12	− 0.39–0.15	0.40
Burn (Chemical)	0.25	− 0.23–0.72	0.31
Burn (Other)	− 0.25	− 1.07–0.57	0.54
BMI	0.00	− 0.01–0.01	0.65
TBSA (%)	0.00	0.00–0.01	0.44
LOHS	0.00	0.00–0.00	0.67
History of anxiety or panic disorder	0.03	− 0.28–0.34	0.84
History of depression	0.38	0.11–0.65	**0.01**
Sex (female)	0.00	− 0.16–0.18	0.94
Age	0.00	− 0.01–0.00	0.12
Ethnicity (Native)	− 0.10	− 0.5343–0.33	0.64
Ethnicity (Black or African American)	0.03	− 0.2739–0.33	0.86
Ethnicity (Hispanic)	0.14	− 0.1058–0.39	0.26
Ethnicity (Asian or Pacific Islander)	− 0.18	− 0.5100–0.15	0.29
Ethnicity (Other)	0.18	− 0.4915–0.85	0.60

*TBSA* total body surface area; *BMI* body mass index; *LOHS* length of hospital stay; *M* mean; *SD* standard deviation; *n* number

Significant p values highlighted in bold font

## Discussion

The present study compares the largest major burns cohort against a general population to provide three major findings: (a) burn injury is associated with chronic pain, anxiety, and depression; (b) TBSA-burned directly correlates with the prevalence of chronic pain, anxiety and depression and (c) history of depression predisposes to post-discharge pain, anxiety, and depression, (d) pain, depression and anxiety are interrelated and may have interactive effects on the process of recovery following burn injury.

### Burn injury is associated with chronic pain, anxiety, and depression

Burn survivors have considerable difficulties in returning to family, social, work, and community roles [2]. The prolonged periods of hospitalization and extensive treatments required in burn patients, particularly those with severe burns, predispose them to psychosocial issues including social isolation, financial challenges, depression, anxiety, post-traumatic stress disorder, and aesthetic dissatisfaction [8]. In this cohort we identified that 82% of burn survivors had at least some pain, 62% had at least some signs of anxiety, and 56% described depression, prevalence values higher than in the normative population (52% pain, 36% anxiety and 46% depression). The prevalence of depression is in line with prior research, with systematic reviews reporting that 54% of burn survivors develop at least “mild” depressive symptoms [9]. Control of burn pain is already recognized as central to the recovery and reintegration of burn survivors [10], while resolution of the psychological problems can improve their quality of life and well-being [11].

Various underlying mechanisms for neuropathic pain have been described including nerve entrapment distal to the injury site caused by edema tracking along the course of nerves, neural adherence and entrapment in hypertrophic post-injury or post-surgical scars, and neuroma formation following iatrogenic transection of cutaneous nerve branches during burn excision [23].

To address both procedural and background pain after burn injury, a comprehensive pain assessment is essential to identify the predominant issue. Establishing a pain treatment plan that incorporates pharmacological and non-pharmacological approaches is crucial. Opioid agonists, particularly long-acting opiates for background pain and short-acting opiates for procedures like wound care, are commonly used. Additionally, supplementation with other drugs such as inhaled nitrous oxide and anxiolytics can be beneficial. Lorazepam has demonstrated effectiveness in alleviating burn pain, particularly by addressing acute anxiety. Non-pharmacological techniques, including cognitive-behavior therapy, hypnosis, and virtual reality distraction have proven efficacy in treating procedural pain [24]. Failure to promptly and adequately address the acute pain and psychological issues that occur during the hospitalization period, on the other hand, can result in a progression to chronic psychiatric morbidities [12].

### TBSA directly correlates with the prevalence of chronic pain, anxiety, and depression

TBSA is found to be independently associated with pain, depression, and anxiety. Evidence from prior studies had not reached a consensus on this correlation. For example, in an analysis of 100 burn patients admitted to a tertiary care private hospital in India Jain et al. found no significant association between TBSA and anxiety or depression [13]. Interestingly, when the authors focused on patients with deep burns a significant association with anxiety and depression could be established. Depth of burn injury has been previously described to be a risk variable in the occurrence of depression, generalized anxiety disorder, and any anxiety disorder [14]. In our study we focused solely on patients with severe burns necessitating surgical treatment, at least > 10%), therefore eliminating the variability of burn depth, and establishing a strong correlation between TBSA and psychosocial challenges. It should be mentioned that even cases of low TBSA warrant particular care, for example burn injuries involving the face, as these patients have been previously shown to be at particular risk of psychosocial compromise reporting significantly higher scores for anxiety and depression than a normative control [25].

### History of depression predisposes to post-discharge pain, anxiety, and depression

A history of depression is found to be independently associated with chronic pain, anxiety, and depression, whereas no association with a history of anxiety was established. It has been previously reported that burn patients with a history of psychiatric disorders are more likely to get burned in the first place, require more surgical procedures, have prolonged hospital stays, experience more dysfunction, and require more assistance [15, 17]. These patients are also at higher risk of suicide, with studies reporting rates of suicide nearly five times greater than the general population [15, 20, 21]. A positive correlation between suicidal ideation and acute pain at discharge has also been described [26]. Awareness of this risk can allow for appropriate screening of at-risk patients and prompt resolution of pain, anxiety and depression in the acute phase and help prevent progression to chronic psychiatric morbidities [12].

Previous research emphasizes the importance of identifying specific ‘red flags,’ such as behavioral disengagement, venting, and self-blame behaviors after burn injury, as triggers for early depression screening and timely intervention [27]. To enhance post-traumatic growth, some studies have found success with interventions promoting positive reframing, the use of religion, social support, and confrontive-coping and acceptance [27, 28].

### Association between smoking and post-discharge pain, anxiety, and depression

Emerging evidence has suggested that smokers with anxiety or depression experience more severe pain and functional impairment, and that this pain in turn induces further motivation to smoke [29]. Increased sensitivity to pain during periods of smoking abstinence has also been shown [30]. In burns patients specifically, smoking has been linked to higher rates of intubation and more frequent infections [31], potentially due to nicotine’s vasoconstrictive effects which predispose to hyperinflammation and subsequent infection [32, 33]. This highlights that although cessation should be encouraged for better outcomes, special care should be taken with optimizing pain management in such patients.

Recognizing the heightened risk of psychological distress among smokers after burn injury underscores the importance of acknowledging their increased susceptibility to burns due to smoking habits. For instance, one study by Carlos et al. highlighted significantly higher rates of burn injuries, morbidity, and mortality in smokers admitted to a community burn unit, particularly associated with home oxygen therapy for chronic obstructive pulmonary disease [34]. About 14.5% of smokers suffering burns from home oxygen therapy had a history of prior injuries from smoking with home oxygen, and about a third of patients had burns extending past 5% TBSA [34]. This emphasizes the need for continuous education and cautious prescription of home oxygen for smokers, underlining the importance of both smoking cessation and effective pain management in optimizing outcomes for this patient population.

### Age and female sex are associated factors for post-discharge pain, anxiety, and depression

A direct correlation between increasing age and prevalence of chronic pain was seen, although age was unrelated to depression or anxiety. At the same time, we establish that women were more likely to experience chronic pain, anxiety, and depression. Female sex has been previously identified as a risk variable predicting anxiety disorders in a study group exposed to two different traumatic events, specifically 128 burn and 55 motor vehicle accident injuries [14]. The higher prevalence of depression in female burn survivors specifically, particularly severe depression, has been previously described, albeit in a low-income country [13]. The exact reasons for this sex difference are not clear although prior research has suggested that women are more prone to depression because of disfigurement [35]. Some hospitals have begun self-esteem building programs targeted at these populations. "Changing Faces" is one example of a proven effective program designed to boost self-esteem in burn patients, mainly geared toward women. It encompasses a hospital-based initiative focusing on image enhancement and social skills, along with a series of publications aimed at assisting patients in coping with various aspects of facial disfigurement [23]. At the same time prior research investigating scarring and distress in children with severe burns identified that female patients had significantly higher sleep disturbances than males [36]. An association between adequate high quality sleep during acute hospitalization and decreased post-burn pain and anxiety has been proposed, which may be one underlying factor for the gender differences in chronic psychosocial challenges [37].

### Pain, depression, and anxiety are interrelated

Our multivariable regression found that the presence of pain and anxiety are independent associated factors for depression. In agreement with this, Cariello et al. used questionnaires to assess the pain, mental health, and daily functioning of 87 outpatient burn patients and found that the presence of pain predicted depression and anxiety [38]. Furthermore, they showed that pain, anxiety, and depression affected ability to work, sexuality and interpersonal relationships. The interrelationship between pain, depression and anxiety and the fact that chronic pain substantially contributes to long-term psychosocial functioning in burn survivors, highlight the value of a multidisciplinary approach that includes mental health professionals during the hospitalization and post-discharge period.

Anti-depressants and anxiety medicines may be a viable option to decrease pain, depression and anxiety. In fact, two studies reported an over 80% response rate to tricyclic antidepressants (TCA) in burn patients with stress and depressive disorders [39, 40]. However, caution is warranted due to the mechanism of TCAs, which inhibit neuronal uptake of norepinephrine and serotonin [41, 42]. While this increases neurotransmitter levels and improves mood, cognition, and anxiety modulation, serotonin also plays a role in wound healing [43]. Thus, the positive response to TCAs may be linked to improved mood symptoms from enhanced wound healing, raising uncertainty about their efficacy for depressive symptoms unrelated to wound progress.

At the same time, prior research has shown that burn patients continue to have high opioid requirement [44]. A high prevalence of burn survivors on opioids lies in contrast to USA efforts to decrease opioid prescribing. Long-term opioid use results in dependence, or opioid use disorder, and possibly other mental health comorbidities. Burn patients with opioid use disorder have been shown to have significantly higher rates of future psychiatric diagnoses, behavioral disturbances, and polysubstance abuse [44]. The study concluded that burn survivors would benefit from an early involvement of pain and mental health experts.

### The effect of glutamine on symptoms of anxiety and depression

Glutamine appeared to affect one component from two of the primary outcomes, that is, glutamine recipients were less likely to feel depressed “a little of the time,” and more likely to feel nervous “some of the time.” However, all other components were insignificant, indicating that there was no overall shift in the response rates within these two questions. It should be noted that prior research has suggested an association between lower glutamine levels and higher depression [45], with supplementation of glutamine proposed as an alternative antidepressant that can increase glutamatergic neurotransmission [46]. As glutamine was only supplemented during the hospitalization period, and our results are not compelling enough, we cannot suggest that this is indeed the case. However, the effects of long-term supplementation of glutamine on depression and anxiety warrant further research.

### Implications for clinical practice

The results of this study add to the prior literature that found burn patients to have a higher predisposition to psychological distress, social isolation, and lower quality of life [47]. Adequate and specialized psychosocial support addressing the various psychological, social, and emotional factors, can enhance patients overall well-being and resilience. Mental health interventions, such as counseling, cognitive-behavioral therapy, and support groups, can help patients cope with the challenges following burn injury, thereby reducing the risk of developing chronic mental health issues. Furthermore, social isolation and loneliness are common among burn survivors, especially during the recovery phase [48]. Building and maintaining strong social support networks can provide emotional comfort, practical assistance, and a sense of belonging, all of which contribute to resilience and can help prevent depression and anxiety. Involving family members and caregivers is also important in the rehabilitation process. Educating and supporting caregivers can enhance their ability to provide effective care, reduce caregiver burden, and improve patient outcomes, thus minimizing the risk for both patients and their caregivers. Peer support programs allow burn survivors to connect with others who have experienced similar challenges. Sharing experiences, advice, and encouragement in a supportive environment can boost morale, increase self-confidence, and foster a sense of community, all of which are protective factors. Providing comprehensive education can empower patients to actively participate in their recovery process. When patients feel informed and empowered, they are more likely to adhere to long-term treatment plans, participate in rehabilitation efforts, and take proactive measures to maintain their long-term physical and mental health. Finally, psychosocial support should extend beyond the acute phase of burn care to encompass long-term follow-up and monitoring. Regular assessments of mental health, social functioning, and quality of life allow healthcare providers to promptly identify and address emerging psychosocial challenges, preventing the progression to a clinical diagnosis in the long term.

### Limitations and future research

All the response data are based on self-reporting, which is inherently associated with inaccuracies. As the questionnaires for each group were conducted following different protocols some of the questions and responses were adjusted to reach a consensus. Furthermore, although the RE-ENERGIZE data span six years (2016–2021) we opted to use only the 2021 NHIS data because the rotating design of the NHIS meant that only 2021 provided data on psychological distress. Furthermore, data prior to 2019 were avoided because the NHIS database was redesigned that year. A further limitation of our study is that the NHIS data is US-based, whereas the RE-ENERGIZE data collection was international, although most patients were based in North America. Propensity score matching was used to minimize this bias and further propensity score matching analysis including only data of US burn patients verified our results. As a limited subset of both populations was sampled, generalizability is also an issue. For example, burn patients were ‘lost to follow-up,’ and comparison of the SF-36 responders to the non-responders identified some significant differences in baseline characteristics. The applicability of our results to other populations, and settings, particularly low- and middle-income countries is uncertain. Given the non-prospective nature of this post hoc analysis some variables which may influence our results, such as the location of the injury, i.e., work or home related, cause of burn (i.e., accidental, self-inflicted or hetero inflicted), body location affected (facial burn), and information on post-discharge return to employment, were unavailable. Furthermore, as we compare a burn cohort to the general population we cannot conclude that burn injury, rather than hospitalization for a severe condition, was responsible for the increased pain, and depression and anxiety symptoms. In a 2021 meta-analysis our group compared the overall health-related quality of life (HRQoL), as assessed by different questionnaires including the SF-36, in three types of critical illness (sepsis, trauma, and burns) and found all three had severely limited HRQoL compared to the general population. The three cohorts did not show substantial differences among each other, which may be attributed to some degree of similarity in the severity of these critical illnesses, the extent of hospitalization, and the related outcomes between the cohorts, or may be due confounding from heterogeneity. A prospectively planned study that compares these three cohorts in terms of pain, anxiety and depression would be a significant contribution to the literature. The biological mechanisms underlying the symptoms of pain, depression and anxiety were beyond the scope of this work. Finally, the cross-sectional design of the study prevents causality inference.

### Main findings

1. Long-term survivors of severe burn injury exhibit pain and symptoms of anxiety and depression significantly more often than the general population, indicating incomplete recovery
2. There is a clear correlation between extent of burn injury and severity of pain and symptoms of anxiety and depression underlining the need for specialized care based on initial trauma severity.
3. Despite > 80% of survivors achieving adequate acute recovery to return home or into rehabilitation pain and symptoms of anxiety and depression remain major problems interfering with everyday life and social reintegration.

### Supplementary Information

**Additional file 1.** Supplemental data tables.

## Data Availability

Datasets generated and analyzed to provide the findings in this study are available from the corresponding author upon reasonable request.
