# Supplementary material for: The long-term intercorrelation between post-burn pain, anxiety, and depression: a post hoc analysis of the “RE-ENERGIZE” double-blind, randomized, multicenter placebo-controlled trial
Source: Crit Care. 2024 Mar 22;28:95. doi: 10.1186/s13054-024-04873-8 (PMC10958907; doi:10.1186/s13054-024-04873-8)
Supplement: Supplementary file 1 — Additional file 1. Supplemental data tables. [file 13054_2024_4873_MOESM1_ESM.docx]

**Supplementary figure 1.** Histogram visualization of the cohorts before and after propensity score matching. The matched groups are balanced, therefore eliminating the initial selection bias. Raw treated: entire burn cohort; Matched treated: matched burn patients; Raw control: entire general population cohort; Matched control: matched general population subjects.


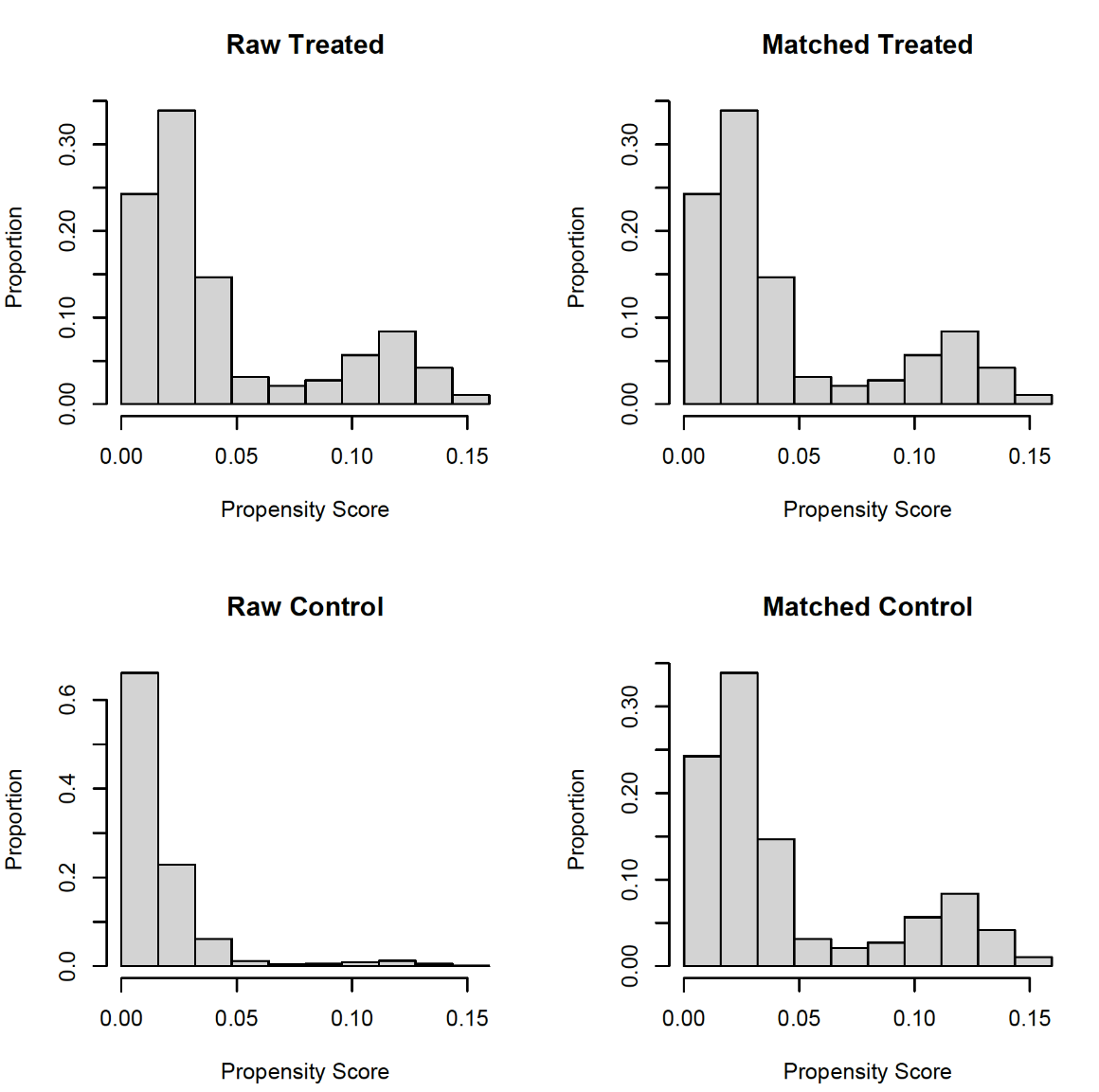


**Supplementary figure 2.** Jitter plot visualization of the cohorts before and after propensity score matching. The matched cohorts show high similarity. Unmatched treated: unmatched burn patients; Matched treated: matched burn patients; Unmatched control: unmatched general population subjects; Matched control: matched general population subjects.
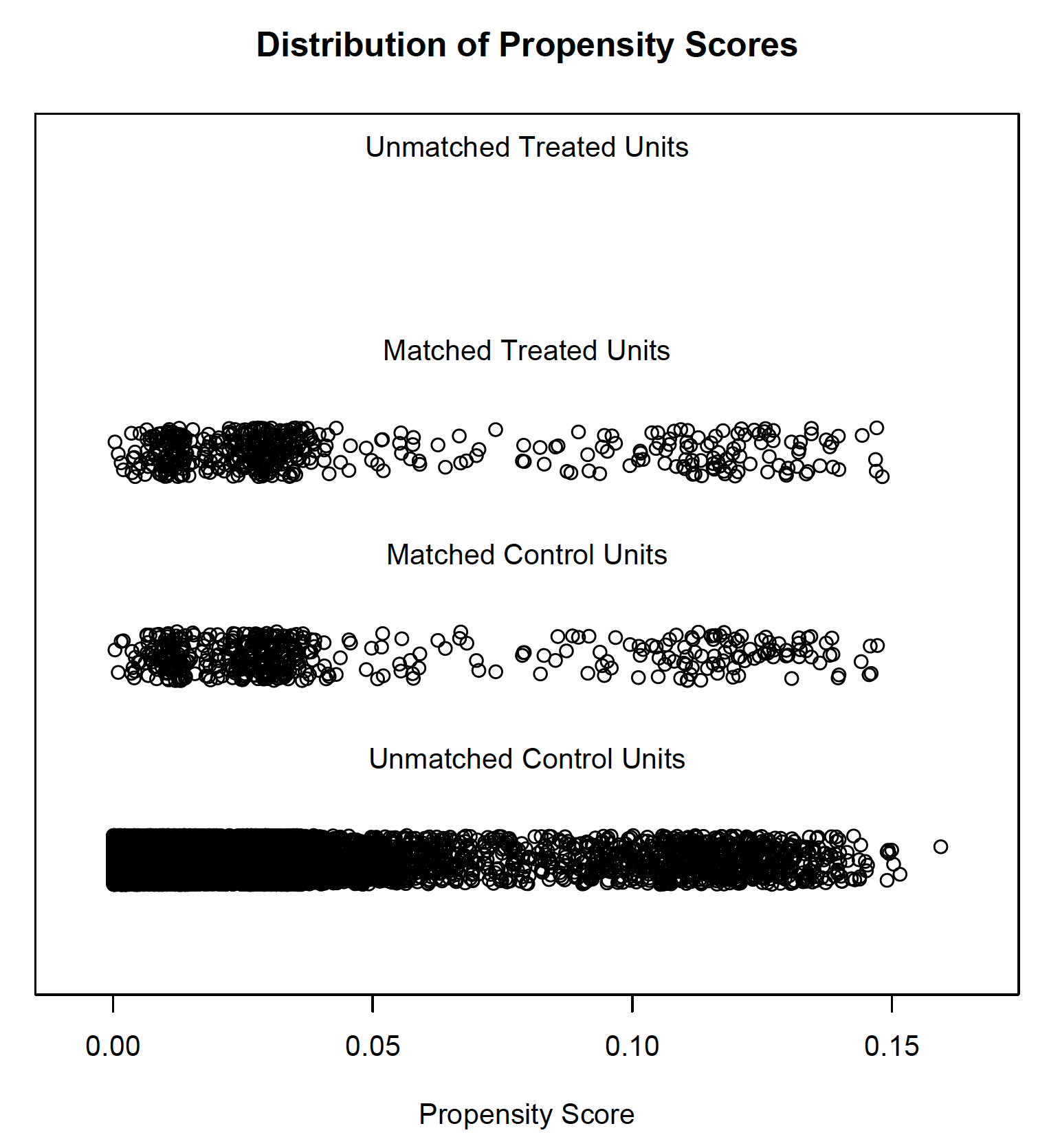


**Supplementary Table 1.** Subject demographics and characteristics with patients stratified by their response to the SF-36 questionnaire. Deceased patients were excluded (n=199). Reported as n (%), unless otherwise stated.

|  | **Responded**  **(n=600)** | **Did not respond (n=401)** | **p value** |
| --- | --- | --- | --- |
| **Sex** |  |  | 0.18 |
| Male | 441 (73.5) | 310 (77.3) |  |
| Female | 159 (26.5) | 91 (22.7) |  |
| **Age in years, M (SD)** | 48.7 (17.1) | 45.0 (17.0) | **0.001** |
| **BMI in kg/m^2^, M (SD)** | 28.3 (6.0) | 27.9 (5.5) | 0.33 |
| **TBSA%, M (SD)** | 30.6 (15.1) | 31.3 (15.1 | 0.45 |
| **Ethnicity** |  |  |  |
| White or Caucasian | 456 (76.0) | 316 (78.8) | 0.32 |
| Native | 18 (3.0) | 5 (1.2) | 0.09 |
| Hispanic | 50 (8.3) | 20 (5.0) | **0.04** |
| Black or African American | 38 (6.3) | 43 (10.7) | **0.02** |
| Asian or Pacific Islander | 31 (5.2) | 11 (2.7) | 0.08 |
| Other | 7 (1.2) | 6 (1.5) | 0.78 |
| **Co-morbidities** | 327 (54.5) | 211 (52.6) | 0.56 |
| **Mental health** |  |  |  |
| Anxiety | 46 (7.7) | 35 (8.7) | 0.56 |
| Depression | 58 (9.7) | 54 (13.5) | 0.07 |
| **Smoker** | 170 (28.3) | 146 (36.4) | **0.01** |
| **APACHE-II score** | 13.5 (7.9) | 13.1 (7.6) | 0.41 |
| **Inhalation injury** | 0 (0.0) | 39 (9.7) | **<0.0001** |
| **Received ventilation** | 321 (53.5) | 200 (49.9) | 0.27 |
| **Received glutamine** | 294 (49.0) | 200 (49.9) | 0.80 |
| **Cause of burn** |  |  |  |
| Chemical | 15 (2.5) | 12 (3.0) | 0.69 |
| Fire | 536 (89.3) | 350 (87.3) | 0.36 |
| Scald | 45 (7.5) | 37 (9.2) | 0.35 |
| Other | 4 (0.7) | 2 (0.5) | >0.99 |
| **Discharge destination** |  |  |  |
| Home | 300 (50.0) | 197 (49.1) | 0.80 |
| Rehabilitation unit | 191 (31.8) | 116 (28.9) | 0.36 |
| Long term care facility | 26 (4.3) | 16 (4.0) | 0.87 |
| Ward in another hospital | 16 (2.7) | 22 (5.5) | **0.03** |
| ACU in another hospital | 9 (1.5) | 7 (1.7) | 0.80 |
| **Length of hospital stay in days, M (SD)** | 42.2 (24.9) | 38.0 (24.5) | **0.01** |
| **Length ICU stay in days, M (SD)** | 38.7 (24.3) | 35.2 (23.3) | **0.03** |

M, Mean; SD, Standard deviation; n, Number; BMI, Body mass index; TBSA, total body surface area; ICU, intensive care unit; ACU, acute care unit.

**Supplementary Table 2.** Subject demographics, characteristics and outcomes following propensity score matching using only patients from the US cohort. Reported as n (%), unless otherwise stated.

|  | **Burn (n=311)** | **Normative (n=311)** | **p value** | |
| --- | --- | --- | --- | --- |
| **Sex (Male)** | 231 (74.3) | 240 (77.2) | 0.45 | |
| **Age in years, M (SD)** | 48.0 (17.5) | 46.2 (16.7) | 0.19 | |
| **BMI in kg/m^2^, M (SD)** | 29.2 (6.4) | 28.5 (8.4) | 0.23 | |
| **Ethnicity** |  |  |  | |
| White or Caucasian | 241 (77.5) | 227 (73.0) | 0.23 | |
| Native | 2 (0.6) | 4 (1.3) | 0.69 | |
| Hispanic | 28 (9.0) | 22 (7.1) | 0.46 | |
| Black or African American | 32 (10.3) | 40 (12.9) | 0.38 | |
| Asian or Pacific Islander | 5 (1.6) | 14 (4.5) | 0.06 | |
| Other | 3 (1.0) | 4 (1.3) | >0.99 | |
| **How much bodily pain have you had?** | | | | |
| None | 46 (14.8) | 149 (47.9) | | <0.0001 |
| A little | 131 (42.1) | 127 (40.8) | | 0.81 |
| Moderate | 84 (27.0) | 14 (4.5) | | <0.0001 |
| A lot | 48 (15.4) | 20 (6.4) | | 0.0004 |
| No response | 2 (0.6) | 1 (0.3) | | >0.99 |
| **How much did pain interfere with your normal work (outside the home or housework)?** | | | | |
| Not at all | 115 (37.0) | 105 (33.8) | | 0.45 |
| A little bit | 68 (21.9) | 41 (13.2) | | 0.006 |
| Quite a bit | 94 (30.2) | 9 (2.9) | | <0.0001 |
| Extremely | 29 (9.3) | 6 (1.9) | | <0.0001 |
| No response | 5 (1.6) | 150 (48.2) | | <0.0001 |
| **How much of the time have you been very nervous?** | | | | |
| All of the time | 14 (4.5) | 1 (0.3) | | 0.001 |
| Most of the time | 24 (7.7) | 4 (1.3) | | <0.0001 |
| Some of the time | 72 (23.2) | 37 (11.9) | | 0.0003 |
| A little of the time | 66 (21.2) | 61 (19.6) | | 0.69 |
| None of the time | 130 (41.8) | 207 (66.6) | | <0.0001 |
| No response | 5 (1.6) | 1 (0.3) | | 0.22 |
| **How much of the time have you felt depressed?** | | | | |
| All of the time | 13 (4.2) | 5 (1.6) | | 0.09 |
| Most of the time | 35 (11.3) | 14 (4.5) | | 0.003 |
| Some of the time | 55 (17.7) | 26 (8.4) | | 0.001 |
| A little of the time | 71 (22.8) | 88 (28.3) | | 0.14 |
| None of the time | 131 (42.1) | 177 (56.9) | | 0.0003 |
| No response | 6 (1.9) | 1 (0.3) | | 0.12 |
| **How much of the time have you felt so down in the dumps that nothing could cheer you up?** | | | | |
| All of the time | 11 (3.5) | 1 (0.3) | | 0.006 |
| Most of the time | 20 (6.4) | 2 (0.6) | | <0.0001 |
| Some of the time | 52 (16.7) | 14 (4.5) | | <0.0001 |
| A little of the time | 53 (17.0) | 27 (8.7) | | 0.003 |
| None of the time | 170 (54.7) | 265 (85.2) | | <0.0001 |
| No response | 5 (1.6) | 2 (0.6) | | 0.45 |
| **How much of the time has your physical health/emotional problems interfered with your social activities?** | | | | |
| All of the time | 38 (12.2) | 3 (1.0) | | <0.0001 |
| Most of the time | 41 (13.2) | 2 (0.6) | | <0.0001 |
| Some of the time | 44 (14.1) | 7 (2.3) | | <0.0001 |
| A little of the time | 57 (18.3) | 0 (0.0) | | <0.0001 |
| None of the time | 127 (40.8) | 299 (96.1) | | <0.0001 |
| No response | 4 (1.3) | 0 (0.0) | | 0.12 |

BMI, Body mass index; M, Mean; SD, Standard deviation; n, Number.

**Supplementary Table 3.** Burn cohort post-discharge pain, anxiety and depression. Reported as n (%), unless otherwise stated.

| **During the past 4 weeks:** | **Burn (n=600)** |
| --- | --- |
| **How much bodily pain have you had?**   \| **How much bodily pain have you had duringthe past 4 weeks?** \| \| --- \| | |
| None | 113 |
| A little | 225 |
| Moderate | 167 |
| A lot | 89 |
| No response | 6 |
| **How much did pain interfere with your normal work (outside the home or housework)?** | |
| Not at all | 221 |
| A little bit | 132 |
| Quite a bit | 178 |
| Extremely | 56 |
| No response | 13 |
| **How much of the time have you felt so down in the dumps that nothing could cheer you up?** | |
| All of the time | 21 |
| Most of the time | 35 |
| Some of the time | 103 |
| A little of the time | 121 |
| None of the time | 303 |
| No response | 17 |
| **How much of the time have you been very nervous?** | |
| All of the time | 27 |
| Most of the time | 54 |
| Some of the time | 153 |
| A little of the time | 134 |
| None of the time | 216 |
| No response | 16 |
| **How much of the time have you felt depressed?** | |
| All of the time | 21 |
| Most of the time | 63 |
| Some of the time | 120 |
| A little of the time | 138 |
| None of the time | 241 |
| No response | 17 |
| **How much of the time has your physical health/emotional problems interfered with your social activities?** | |
| All of the time | 52 |
| Most of the time | 92 |
| Some of the time | 111 |
| A little of the time | 104 |
| None of the time | 226 |
| No response | 15 |

**Supplementary Table 4.** Normative cohort pain, anxiety and depression. Reported as n (%), unless otherwise stated.

| **During the past 3 months:** | **Normative (n=24666)** |
| --- | --- |
| **Thinking about the last time you had pain, how much pain did you have?** | |
| None | 9866 |
| A little | 7615 |
| Moderate | 6204 |
| A lot | 2520 |
| No response | 14 |
| **Over the past three months, how often did your pain limit your life or work activities?** | |
| Not at all | 8697 |
| A little bit | 5675 |
| Quite a bit | 932 |
| Extremely | 1035 |
| No response | 14 |
| **How much of the time have you felt so down in the dumps that nothing could cheer you up?** | |
| All of the time | 158 |
| Most of the time | 409 |
| Some of the time | 1679 |
| A little of the time | 3115 |
| None of the time | 20846 |
| No response | 459 |
| **How much of the time have you been very nervous?** | |
| All of the time | 434 |
| Most of the time | 746 |
| Some of the time | 3662 |
| A little of the time | 6113 |
| None of the time | 15245 |
| No response | 466 |
| **How much of the time have you felt depressed?** | |
| All of the time | 979 |
| Most of the time | 1557 |
| Some of the time | 2002 |
| A little of the time | 7700 |
| None of the time | 14004 |
| No response | 56 |
| **How much of the time has your physical/emotional health interfered with your social activities?** | |
| All of the time | 364 |
| Most of the time | 529 |
| Some of the time | 1467 |
| A little of the time | Not asked |
| None of the time | 24286 |
| No response | 20 |

**Supplementary Table 5.** Pain, anxiety and depression in patients who received glutamine versus placebo. Reported as n (%), unless otherwise stated.

|  | **Glutamine (n=294)** | **Placebo (n=306)** | **p value** | |
| --- | --- | --- | --- | --- |
| **How much bodily pain have you had?** | | | | |
| None | 58 (19.7) | 55 (18.0) | | 0.60 |
| A little | 109 (37.1) | 116 (37.9) | | 0.87 |
| Moderate | 84 (28.6) | 83 (27.1) | | 0.72 |
| A lot | 40 (13.6) | 49 (16.0) | | 0.42 |
| No response | 3 (1.0) | 3 (1.0) | | >0.99 |
| **How much did pain interfere with your normal work (outside the home or housework)?** | | | | |
| Not at all | 111 (37.8) | 110 (35.9) | | 0.67 |
| A little bit | 64 (21.8) | 68 (22.2) | | 0.92 |
| Quite a bit | 91 (31.0) | 87 (28.4) | | 0.53 |
| Extremely | 22 (7.5) | 34 (11.1) | | 0.16 |
| No response | 6 (2.0) | 7 (2.3) | | >0.99 |
| **How much of the time have you been very nervous?** | | | | |
| All of the time | 9 (3.1) | 18 (5.9) | | 0.12 |
| Most of the time | 26 (8.8) | 28 (9.2) | | >0.99 |
| Some of the time | 86 (29.3) | 67 (21.9) | | **0.04** |
| A little of the time | 64 (21.8) | 70 (22.9) | | 0.77 |
| None of the time | 103 (35.0) | 113 (36.9) | | 0.67 |
| No response | 6 (2.0) | 10 (3.3) | | 0.45 |
| **How much of the time have you felt depressed?** | | | | |
| All of the time | 12 (4.1) | 9 (2.9) | | 0.51 |
| Most of the time | 34 (11.6) | 29 (9.5) | | 0.43 |
| Some of the time | 60 (20.4) | 60 (19.6) | | 0.84 |
| A little of the time | 54 (18.4) | 84 (27.5) | | **0.01** |
| None of the time | 125 (42.5) | 116 (37.9) | | 0.28 |
| No response | 9 (3.1) | 8 (2.6) | | 0.81 |
| **How much of the time have you felt so down in the dumps that nothing could cheer you up?** | | | | |
| All of the time | 11 (3.7) | 10 (3.3) | | 0.83 |
| Most of the time | 18 (6.1) | 17 (5.6) | | 0.86 |
| Some of the time | 56 (19.0) | 47 (15.4) | | 0.24 |
| A little of the time | 54 (18.4) | 67 (21.9) | | 0.31 |
| None of the time | 147 (50.0) | 156 (51.0) | | 0.87 |
| No response | 8 (2.7) | 9 (2.9) | | >0.99 |
| **How much of the time has your physical health/emotional problems interfered with your social activities?** | | | | |
| All of the time | 20 (6.8) | 32 (10.5) | | 0.15 |
| Most of the time | 52 (17.7) | 40 (13.1) | | 0.14 |
| Some of the time | 57 (19.4) | 54 (17.6) | | 0.60 |
| A little of the time | 48 (16.3) | 56 (18.3) | | 0.59 |
| None of the time | 112 (38.1) | 114 (37.3) | | 0.89 |
| No response | 5 (1.7) | 10 (3.3) | | 0.30 |
